# Let the records speak: an exploration of rehabilitation services offered in primary healthcare, Johannesburg metropolitan district

**DOI:** 10.1186/s12913-024-10965-6

**Published:** 2024-04-22

**Authors:** Lebogang Maseko, Fasloen Adams, Hellen Myezwa

**Affiliations:** 1 Occupational Therapy Department, School of Therapeutic Sciences, Faculty of Health Sciences, University of the Witwatersrand, Johannesburg, Gauteng, South Africa; 2 Department of Health and Rehabilitation Sciences, Division of Occupational Therapy, Faculty of Medicine and Health Sciences, Stellenbosch University, Stellenbosch, Western Cape South Africa; 3 Physiotherapy Department, School of Therapeutic Sciences, Faculty of Health Sciences, University of the Witwatersrand, Johannesburg, Gauteng, South Africa

**Keywords:** Rehabilitation, Delivery of health care, Integrated services, Speech therapy, Occupational therapy, physiotherapy, audiology, Community health centres, Patient-centred care, Health services for persons with disabilities

## Abstract

**Background:**

Primary healthcare in South Africa aims to transform the national health system by emphasising community-based care and preventive strategies. However, rehabilitation services, particularly for individuals with disabilities and chronic non-communicable diseases, are often overlooked in primary healthcare. This study aimed to investigate the provision of primary healthcare rehabilitation services in the Johannesburg Metropolitan District by exploring client sociodemographics and variations in services provided by rehabilitation professionals.

**Methods:**

A retrospective review of clinic rehabilitation records from 2011 to 2020 was conducted at nine provincially funded community health centres (CHCs) offering rehabilitation services. Stratified sampling facilitated record selection based on rehabilitation service type and year. A specifically designed data extraction tool captured demographics, disabilities, rehabilitation received, and referral sources. Descriptive analysis used means, standard deviations, and frequencies.

**Results:**

The findings show a diverse client population with a wide age range, with a significant proportion falling into the < 5 years and 30–49 years age groups. Neuromusculoskeletal and movement-related disabilities were most prevalent, affecting approximately two-thirds of clients. Referral sources were often undocumented, and inconsistent discharge information with no record of patient follow up, highlighted the need for improved documentation practices. Clinic visits were the primary service delivery mode, followed by limited home visits and outreach services. Occupational therapy and physiotherapy were the most used services. Speech and language therapy services were underused, and some CHCs lacked audiology services. There were variations in the number of individual and group sessions provided by the different rehabilitation services, and there were age- and disability-specific differences in service use.

**Conclusion:**

This study offers insights into rehabilitation service provision in the Johannesburg Metropolitan District and enhances our understanding of rehabilitation services in primary healthcare settings. It underscores the importance of a multidisciplinary rehabilitation team to address diverse rehabilitation needs, improving documentation and discharge practices, expanding service delivery models, and reducing disparities in service use. The findings inform strategies for optimising service delivery, workforce, resource allocation, and intersectoral collaboration to ultimately enhance the quality and accessibility of integrated rehabilitation services.

## Background

International studies consistently show that increased investment in a broad range of rehabilitation services yields substantial health, social, and economic benefits [1]. Ensuring the provision of rehabilitation services to the entire population is considered a crucial aspect of achieving universal health coverage (UHC) [2]. The rehabilitation services contribute to community participation and wellbeing by improving capacity, functioning, and modifying the environment of people suffering from a wide range of health conditions [3]. Moreover, the provision of services using a primary healthcare (PHC) approach for optimal health, is achieved through meeting people’s health needs and preferences as close as possible to their everyday environment. This encompasses a range of healthcare activities, along the continuum of health care including promotion, prevention, treatment, rehabilitation, and palliative care. The PHC approach has been shown to reduce hospital admissions, healthcare costs, and promote community development [4].

There is international recognition of the importance of rehabilitation in health systems supporting first-contact and providing accessible, continued, comprehensive and coordinated patient-focused primary health care. However, the integration of rehabilitation services into health systems has progressed slowly in low- and middle-income countries [5]. A mere 25% of countries in the West Pacific region of the World Health Organisation (WHO) have sustainable PC rehabilitation services [6]. Fragmented healthcare and inadequate integration of rehabilitation into comprehensive health services are also reported in Iran and Nigeria in the African region [7, 8]. Consequently, individuals with disabilities, the ageing population, and those burdened with chronic communicable and non-communicable diseases face inadequate access to rehabilitation services at PC level [5]. Recognising the need for solutions and practical guidance on integrating rehabilitation in PHC is highlighted by the WHO in their Rehabilitation 2030 initiative [9], and the Astana declaration on PHC and rehabilitation [10].

The Joint Learning Network for Universal Health Coverage emphasises the significance of raising awareness, capacity-building, and providing adequate data about services [11]. This action is supported, in the context of the WHO vision for primary care which also recognises the importance of functioning as a third health indicator [2]. Therefore, the data collected for our study incorporated the concept of function defined by the body functioning domains of the International Classification of Functioning, Disability and Health (ICF) [12].

In South Africa, PHC plays a pivotal role in the healthcare system as proposed by government policies, including the White Paper for the Transformation of the Health System [13] and the National Health Act of 2003 [14], which underscore the significance of the PHC approach as the cornerstone of the country’s healthcare transformation. Although the commitment to the PHC approach is reinforced by the National Health Insurance (NHI) Bill of July 2019, adopted in November 2023 [15], the current recognition of PC in South Africa is predominantly rooted in a medical model philosophy. The medical model emphasises nurse-driven services with support from doctors, pharmacists, and dentists, while rehabilitation services and person-centred care are not adequately encompassed or prioritised [16, 17]. Efforts to address deficiencies in PHC facilities in South Africa are outlined in the ‘Ideal Clinic’ programme. However, the programme does not explicitly address the staffing or resource allocation for rehabilitation services, apart from a general acknowledgment of the significance of providing access to rehabilitation services for patients [18, 19]..

The provision of multidisciplinary rehabilitation depends on the availability of skilled personnel, and these professions include occupational therapy, physiotherapy, physical and rehabilitation medicine, prosthetics and orthotics, psychology, social work and speech and language therapy [20]. In South Africa, PC rehabilitation services are only offered by rehabilitation professionals such as occupational therapists, physiotherapist, speech and language therapist and audiologists as professionally designated by the Health Professionals Council of South Africa (HPCSA). The rehabilitation services are far from optimal in that they are mainly hospital based with little consideration of broad multi-, inter-and trans- disciplinary teams [21] that can contribute to health care in conjunction with those registered as allied health services [22]. Other health professionals in shortage at the primary care level include nurses, psychologists, prosthetists and orthotists, social workers, and doctors. Therefore, the implementation of intersectoral rehabilitation services in PHC facilities remains limited in South Africa with less than a quarter of facilities currently providing such services [23, 24].

In contrast, PHC rehabilitation research in high-income countries indicates that occupational therapy, physiotherapy and speech and language therapy services are well established. Studies show that these services primarily focus on chronic disease, self-management and improving physical functioning, which is often supplemented with online support for clients [25]. In Sweden, PC physiotherapists predominantly provide physical activity programmes to middle-aged and older adults with conditions such as musculoskeletal disorders, obesity, hypertension, and diabetes [26]. Occupational therapists practicing in PC settings in Norway report that over 80% of their clients are adults and 44% are old age pensioners. The study reported that the conditions treated by occupational therapists included neurological diseases, particularly stroke, as well as musculoskeletal and mental illnesses, which accounted for 13–20% of referrals. Notably, 96% of clients presented with movement impairments, and 94% of them experience challenges in activities and participation related to education, work, and leisure [27]. In the UK, the duration of physiotherapy intervention and referral patterns in PC indicate that services may be offered for less than a month. However, most clients receive supportive treatment over a two-year period, with many referrals originating from triage at the clinic reception or through self-referral [28]. A Norwegian study indicates physiotherapy services for smaller numbers of children in PHC clinics where most clients present with motor development impairments and lower limb orthopaedic diagnoses [27]. Additionally, clients with prematurity and heart or lung disease also receive PC rehabilitation services [29].

In low to middle income countries such as Brazil, referrals for speech and language therapy PC services are mainly for children. They treat learning disorders, poor language development, Attention Deficit Hyperactivity Disorder, stuttering, mutism, and hearing issues [30]. A study from Indonesia noted a higher percentage of patients with neurological conditions receiving treatment [31]. A study conducted in South Africa highlights that clients who receive physiotherapy services at PHC clinics most commonly present with back pain, stroke, and upper and lower limb injuries [32]. However, despite this information, there is a critical lack of supporting evidence in South Africa regarding the use of rehabilitation services at a PC level and the extent to which they are integrated into PHC [24].

In South Africa sociodemographic data concerning the individuals who use PC rehabilitation services, the specific rehabilitation professionals who provide these services, and the services provided are scarce. The WHO Rehabilitation Programme [33] acknowledges the importance of gathering information on existing services and the sociodemographic information of the people accessing these services to inform the development of comprehensive rehabilitation interventions at both national and sub-national levels to effectively meet the individual needs of diverse populations and boost rehabilitation planning efforts [24, 33].

Therefore, the objective of the current study was to investigate the extent and nature of rehabilitation services provided in the Johannesburg Metropolitan District. The study specifically focussed on examining the structure, accessibility, delivery, and use of these services to address this knowledge gap.

## Methods

A retrospective review of clinic rehabilitation records was conducted in an urban setting to investigate the rehabilitation services provided between 2011 and 2020. The research setting was the Johannesburg Metropolitan District, which encompasses 125 PC clinics and CHCs across seven regions, serving a population of 5.5 million [34]. Of the 5.5 million, 2.35 million have a monthly income that falls below the upper limit poverty line of ZAR 1227 (estimated $61) per person [35]. Only nine of the 125 PC clinics and CHCs in the Johannesburg Metropolitan District are managed by the provincial rather than municipal health services. These nine facilities provide rehabilitation services and were selected for our study. The rehabilitation services encompass individual and group outpatient therapy, community reintegration as a focus, outreach and home visits, as well as education and awareness campaigns facilitated by multidisciplinary teams [36].

Stratified sampling was used to select rehabilitation service paper-based records according to the only rehabilitation service providers employed at the clinics: occupational therapists, physiotherapists, speech and language therapists and audiologists. Within each discipline, further stratification was performed based on the year of the records available. The sample size was determined using Cochran’s formula [37], setting a 95% confidence level and a 5% margin of error. There were 238 740 records spanning a 9-year period, and therefore, a review of at least 540 records was deemed necessary. Although the period 2011–2020 was initially identified for the record review, not all facilities had rehabilitation service user records for those years because of varying record storage practices. Therefore, the sampling included client rehabilitation paper-based records for the years available at each facility ranging from 2011 to 2020, inclusively. A random sample was extracted from the records stratified for each rehabilitation service and year from the available records at each facility to fulfil the required sample size.

Data extraction was done using a data extraction tool designed and piloted by the first author in Microsoft Excel. The tool facilitated the collection of various data points, including sociodemographic information such as age and disability related to impairment, which was classified using the classification of the ICF based on body functioning domains [12]. Additionally, data regarding the referral source and nature and duration of received rehabilitation and of services provided by rehabilitation discipline, were extracted. Permission to conduct the study was obtained from the Gauteng Department of Health and the Human Research Ethics Committee (Medical) of the Faculty of Health Sciences at the University of the Witwatersrand (M190466). Descriptive analysis was used to analyse the collected data using means, standard deviations, and frequencies to describe the profile of the service users and the characteristics of the rehabilitation services they received.

## Results

A total of 630 paper-based records were reviewed. The number of records extracted varied across the clinics due to the differences in the availability of records; Lenasia Clinic had the highest availability (13.5%) of records, and the Hillbrow CHC had the lowest (7.0%) (Table 1).

**Table 1:** Distribution of records extracted at each clinic or CHC (N = 630)

Clinic	n	%
Alexandra CHC	81	12.9
Hillbrow CHC	44	7.0
Stretford CHC	54	8.6
Lenasia CHC	85	13.5
Lenasia South CHC	79	12.5
Mofolo CHC	69	11.0
Zola CHC	63	10.0
Discoverers CHC	77	12.2
Chiawelo CHC	78	12.3
Total	630	100

### Sociodemographic profile of clients

The analysis of client sociodemographics revealed that the mean age at the time of assessment was 32 years (SD = 35.2) and ranged from 0 to 102 years. Most clients fell into the age groups < 5 years (22.9%) and 30–49 years (24.1%). Older participants constituted only 11% of the sample (Table 2).

**Table 2:** Age of clients (N = 630)

Age at assessment (years)	n	%
< 5	144	22.9
5–14	94	14.9
15–29	54	8.6
30–49	152	24.1
50–64	117	18.6
65 and above	69	11.0

### Disability related to impairments (ICF body function)

Regarding affected functioning categorized by the WHO ICF domains of body function, neuromusculoskeletal and movement-related functions (b7) were the most common and occurred in approximately two-thirds of clients (66.2%), followed by mental function impairments (b1) (15.6%).

However, only 4.4% of clients were recorded as having multiple impairments although most clients had multiple functioning domains affected (Table 3).

**Table 3:** Distribution of types of impairments (ICF body function and codes) (N = 630)

ICF body function and codes	n	%*
Neuromusculoskeletal and movement-related functions (b7)	417	66.2
Mental functions (b1)	98	15.6
Voice and speech functions (b3)	57	9.1
Sensory functions and pain (b2)	65	10.3
Functions of the cardiovascular, haematological, immunological, and respiratory systems (b4)	10	1.6
Genitourinary and reproductive functions (b6)	1	0.2
Functions of the digestive, metabolic, and endocrine systems (b5)	2	0.3
Functions of the skin and related structures (b8)	5	0.8
**Number of impairments**		
Single	602	95.6
Multiple	28	4.4

*Percentages do not add up to 100 because some clients had multiple impairments

### Rehabilitation services received

#### Source of referral

Clients were referred to the PC facility from various sources. However, the referral source for most clients (61.4%) was not recorded. Among the recorded source of referrals, most were down referrals from secondary and tertiary hospitals. Referrals from other sources include NGOs, private doctors, and other services at the PC level (Table 4).

**Table 4:** Source of referral (N = 630)

Source of referral	n	%
Hospital	164	26.0
Self	39	6.2
Teacher	14	2.2
Unknown	387	61.4
Other	26	4.1

### Reasons for discontinued therapy

Discharge information was inadequately reported in the records, but most clients were lost to follow-up (64.1%), and 15.1% were referred elsewhere. (Table 5).

**Table 5:** Reasons for discontinued therapy (N = 630)

Reasons for discontinued therapy	n	%
Loss to follow-up	404	64.1
Deceased	2	0.3
Referred elsewhere	95	15.1
Unknown	46	7.3
Discharged	83	13.2

### Number and types of visits for all rehabilitation services

Clinic visits, where clients attend outpatient rehabilitation services, accounted for the largest number of visits, ranging from one to six or more, with most clients only seen once (48.7%). Six home visits to clients, and eight outreach visits were recorded where clients were at school or a community group. (Table 6).

**Table 6:** Number and types of visits for all rehabilitation services (N = 630)

Number and type of visit (n = 630)	n	%
**Number of clinic visits by clients**		
1	307	48.7
2–3	214	34.0
4–5	68	10.8
6+	41	6.5
	**Mean (SD)**	**Range**
	2.3 (2.1)	1–15
**Number of home visits to clients**		
1	6	1.0
**Number of outreach visits to clients**		
1	4	0.6
2	1	0.2
3	1	0.2
5	2	0.3

### Rehabilitation services received and frequency of sessions

Occupational therapy and physiotherapy were the most frequently provided rehabilitation services across the nine facilities at more than 40%, while audiology had the lowest use (7.1%). It is important to note that many clients attended multiple therapies (Table 7).

**Table 7:** Distribution of rehabilitation services received and frequency of sessions (N = 630)

Rehabilitation services received	n	%*
Occupational therapy	261	41.4
Physiotherapy	253	40.2
Speech and language therapy	151	24.0
Audiology	45	7.1

*Percentages do not add up to 100 because clients attended multiple therapies

### Individual and group sessions received

The records indicated that most clients received at least one individual or group session from specific rehabilitation services (ranging from 87 to 100%). The mean number of individual sessions varied across services, with occupational therapy and speech and language therapy averaging 2.5 sessions and audiology averaging 1.7 sessions. The number of individual sessions ranged from 1 to 13 for occupational therapy, 1 to 12 for physiotherapy and speech and language therapy, and 1 to 6 for audiology (Table 8).

**Table 8:** Individual and group sessions received (N = 630)

Individual sessions	n	%	Group Sessions	n	%
No. of occupational therapy sessions	204		No. of occupational therapy sessions	28	
1	87	43.0	1	18	64.3
2–3	76	37.2	2	7	25
4–5	26	13.0	3	1	3.6
6–13	15	7.4	5	2	7.1
	Mean (SD)	Range		Mean (SD)	Range
	2.5 (2.1)	1–13		7.00 (7.78)	18–1
No. of physiotherapy sessions	212		No. of physiotherapy sessions	23	
1	116	55.0	1	19	82.6
2–3	75	35.4	2	3	13.0
4–5	14	6.6	3	1	4.3
6–12	7	3.3			
	Mean (SD)	Range		Mean (SD)	Range
	1.9 (1.6)	1–12		5.75 (8.98)	19–1
No. of speech and language therapy sessions	123		No. of speech and language therapy sessions	12	
1	58	47.2	1	8	66.7
2–3	34	27.6	2	3	25
4–5	21	17.1	5	1	8.3
6–12	10	8.1			
	Mean (SD)	Range		Mean (SD)	Range
	2.5 (2.2)	1–12		1.33 (1.52)	8–1
No. of audiology sessions	41		No. of audiology sessions	39	
0–1	27	65.9	1	39	100
2–3	10	24.0			
4–5	3	7.3			
6+	1	2.4			
	Mean (SD)	Range		Mean (SD)	Range
	1.7 (1.3)	1–6		n/a	n/a

### Rehabilitation provided per clinic

No significant difference was found between occupational therapy and physiotherapy services across the nine clinics. However, speech and language therapy was provided to less than 20% of total clients at only four of the nine CHCs. Four CHCs did not offer audiology services (Table 9).

**Table 9:** Rehabilitation sessions per discipline received by clinic (N = 630)

Clinic	Occupational therapy	Physiotherapy	Speech and language therapy	Audiology		
	n (%) *				Chi squared	P value
Alexandra CHC (n = 99)	31 (31.9)	29 (29.9)	23 (21.6)	16 (16.4)	8.51	0.121
Hillbrow CHC (n = 51)	22 (43.1)	12 (23.5)	16 (31.4)	1 (2.0)	16.37	0.001
Stretford CHC (n = 54)	21 (38.9)	23 (42.6)	10 (18.5)	0 (0.0)	9.81	0.007
Lenasia CHC (n = 94)	30 (31.9)	30 (31.9)	25 (26.6)	9 (9.6)	12.94	0.004
Lenasia South CHC (n = 83)	31 (37.3)	28 (33.7)	10 (12.0)	14 (16.9)	18.32	0.004
Mofolo CHC (n = 93)	37 (39.8)	30 (32.3)	26 (28.0)	0 (0.0)	2.24	0.326
Zola CHC (n = 67)	33 (49.3)	33 (49.3)	1 (1.5)	0 (0.0)	45.17	0.000
Discoverers CHC (n = 88)	32 (36.4)	35 (39.8)	16 (18.2)	5 (5.7)	26.78	0.000
Chiawelo CHC (n = 81)	24 (29.6)	33 (40.7)	24 (29.6)	0 (0.0)	2.39	0.301

*n for sessions is greater than the number of sessions as some patients were seen in individual and group sessions

### Rehabilitation received per discipline by disability related to body function

The analysis of client demographics and disabilities in relation to rehabilitation services revealed that clients with mental function disabilities were predominantly seen by occupational therapists. Physiotherapists primarily worked with clients with movement-related dysfunction while speech and language therapists treated those with voice and speech impairments associated with neuromusculoskeletal disabilities.

As expected, audiologists saw a higher proportion of clients with sensory function and pain-related disabilities (Table 10).

**Table 10:** Rehabilitation received per discipline by disability (ICF body function and codes) (N = 630)

Disability (ICF body function)*	Occupational therapy	Physiotherapy	Speech and language therapy	Audiology		
	n (%)				Chi square	P value
Neuromusculoskeletal and movement-related functions (n = 469) (b7)	169 (40.5)	241 (57.8)	58 (14.0)	1(0.2)	26.14	0.001
Mental functions (n = 118) (b1)	86 (87.8)	9 (9.2)	22 (22.5)	1 (1.0)	155.19	0.001
Voice and speech functions (n = 67) (b3)	9 (15.8)	3 (5.3)	51 (89.5)	0 (0.0)	115.51	0.001
Sensory functions and pain (n = 79) (b 2)	9 (14.0)	3 (4.6)	23 (35.3)	44 (67.7)	77.01	0.001
Multiple disabilities (n = 41)	25 (89.3)	9 (32.1)	6 (21.4)	1 (3.6)	112.09	0.001

*n for disability (body function) is greater than the number of cases as some patients were seen by more than one discipline

### Rehabilitation received per discipline by age at assessment

A significant difference was observed in the percentage of clients seen in different age groups across all four rehabilitation services. A significantly greater percentage of younger age groups, including children and adolescents, received occupational therapy and speech and language therapy, while young, middle, and older adults received services from physiotherapy and audiology services (Table 11).

**Table 11:** Rehabilitation received per discipline by age at assessment

Age at assessment*	Occupational therapy	Physiotherapy	Speech and language therapy	Audiology	Chi square	P value
	n (%)					
< 5 (n = 175)	91 (63.2)	23 (15.97)	60 (41.7)	1 (0.7)	74.27	0.001
5–14 (n = 106)	50 (53.2)	11 (11.7)	43 (45.7)	2 (2.1)	48.36	0.001
15–29 (n = 57)	24 (44.4)	27 (50.0)	3 (5.6)	3 (5.6)	35.84	0.001
30–49 (n = 163)	44 (29.0)	88 (57.89)	17 (11.2)	14 (9.2)	55.81	0.001
50–64 (n = 163)	33 (28.2)	68 (58.12)	15 (12.8)	13 (11.1)	55.20	0.001
65+ (n = 80)	19 (27.5)	36 (52.17)	13 (18.8)	12 (17.4)	18.50	0.003

*n for age at assessment is greater than the number of patients as some patients were seen by more than one discipline

## Discussion

The review of client records across the nine PC facilities providing rehabilitation services in the Johannesburg Metropolitan District revealed the sociodemographic profile of clients accessing rehabilitation services, and indicated a wide age range, with a mean age of 32 years. The high proportion of clients in the < 5 years and 30–49 years age groups suggests that rehabilitation services are used by both children and adults within the district, while the older adults constituted a smaller portion of the sample. The wide age range of rehabilitation service users is encouraging, thus rehabilitation PC services should be available across the life course of service users, along the continuum of care, and reflective of all types of care required in the healthcare system in trying to achieve UHC [4]. The age distribution is different to trends in Europe and the UK where most clients are older adults [27–29]. The age distribution aligns with the diverse population demographics of the Johannesburg Metropolitan District [38] where, services for children under the age of 5 years and the older adults are free of charge in South African clinics, which may contribute to the observed age distribution.

Both the Department of Education and the Department of Health share responsibilities for children over the age of six, with the Department of Education specifically accountable for learning and developmental interventions [39]. Furthermore, the high proportion of children under 5 years is aligned with population-based data that shows that the proportion of children with disabilities in low- and middle-income countries is high [40, 41]. It would further explain the high proportion of paediatric neuromusculoskeletal, and movement-related functions (b7) seen in the CHCs in this study. The records reviewed in our study indicate that over 33% of clients receiving services were children and adolescents, compared to 20% reported in a high income country such as Norway [27]. The findings are congruent with the South African statistics indicating nearly 30% of the population is under the age of 15 years [42].

Thus, services must be configured to optimise outcomes for clients across different life stages according to the specific national context to ensure that age-specific rehabilitation needs are adequately addressed in the planning and delivery of services.

Among the clients accessing rehabilitation services, neuromusculoskeletal and movement-related functions were the most prevalent disabilities, followed by mental function (b1) disabilities. These findings are consistent with global trends, emphasising the high prevalence of musculoskeletal and neurological conditions and the importance of addressing mental health within rehabilitation services [26, 43, 44]. This aligns with international studies where physiotherapists and occupational therapists treating adults confirmed they most frequently provide interventions for clients with these conditions using a PHC approach [45]. Mental health, which is rarely mentioned in relation to PC rehabilitation, can be considered in two categories, namely individuals with mental health conditions as a diagnosis, and those with physical impairments who experience associated mental health concerns [2]. The presence of multiple impairments in a subset of clients further highlights the complexities present in rehabilitation service users and underscores the need for comprehensive and interdisciplinary approaches to rehabilitation [46–48] that excel in a wide range of generalist competencies rather than specialised or narrow competencies.

Our study indicated poor recording of the referral sources for most clients which represents a substantial data gap. It hinders the understanding and formalising of the referral pathways through which clients access rehabilitation services and impedes efforts to improve coordination and continuity of care required to provide integrated rehabilitation services in PHC [49]. Enhancing data collection practices to capture this information uniformly and accurately would provide valuable insights into care coordination and identify potential areas for intervention. The prevalence of hospital down referrals suggests that collaboration between PC facilities and higher-level healthcare institutions is essential in facilitating access to rehabilitation services [49]. It could be, however, that overstretched hospital based services may account for an influx of patients referred to PC rehabilitation services who still require acute in-hospital care, but are down-referred due to high patient numbers in hospitals and increased demand for in-patient beds [50].

Discharge information was inadequately reported in the records, with most clients being lost to follow-up. While hospitals are identified as the primary source of down referrals, the absence of documented discharge information at the PC level raises concerns regarding the extent of collaborative referrals and feedback on referrals, particularly for clients with complex diseases. This finding highlights a gap in record-keeping practices and long-term follow-up of clients. It emphasises the need for strategies to improve accessible record keeping, post-rehabilitation support, and long-term management in PC. Understanding the reasons for discharge and lack of treatment continuity is essential. Ways to enhance patient engagement and adherence to rehabilitation programmes are essential for achieving optimal outcomes and should be explored[51]. Addressing accessible and comprehensive record keeping and long-term follow-up is important for evaluating the effectiveness of interventions and providing appropriate support to clients throughout their rehabilitation journey.

Clinic visits were found to be the most common mode of service delivery, with limited home visits and outreach visits to NGOs. This suggests that the current focus of rehabilitation services primarily revolves around clinic-based care, with limited attention given to delivering services in clients’ homes, care institutions or reaching out to underserved communities [52]. The literature highlights alternative models of care, such as community-based rehabilitation and the use of community health workers in areas with limited access to PC rehabilitation services [53]. Expanding service delivery beyond PHC clinic settings through outreach services and home-based rehabilitation programmes could enhance accessibility for vulnerable populations and ensure a more comprehensive approach to rehabilitation. Although tele-rehabilitation may be an option this is impacted by high data costs and lack of constant electricity supply in South Africa [54].

Increasing investment in rehabilitation is important for expanding the workforce and capacity in South Africa. The investment includes funding human resources for primary care rehabilitation services. Such services should be integrated into multidisciplinary ward-based outreach teams located in primary care facilities. These teams would provide a range of services—outreach, preventive, promotive, curative, rehabilitative, and palliative—to individuals, groups, and communities. Additionally, fostering partnerships between government and NGOs is essential to enhance these efforts. [55, 56]. Moreover, alternative service delivery models have the potential to improve the reach and effectiveness of rehabilitation services and enhance their integration into PHC. Community-based rehabilitation with an emphasis on community participation and empowerment has been shown to improve health outcomes [53]. Trained community health workers can serve as liaisons between the healthcare system and the community by building trust and establishing open communication channels between rehabilitation providers and users [57].

The frequency of clinic visits and the number of group sessions also varied slightly between the different rehabilitation services, with a small percentage of clients receiving six to 13 individual sessions or more than five group sessions. This is a concern considering the chronic nature of some conditions receiving intervention at clinics. Research in the UK indicates that most PC physiotherapy clients are seen for at least two years for initial rehabilitative and later supportive care [28]. The poor records, loss to follow-up with limited resources in the clinics, and clients experiencing barriers in terms of transport and finances to access clinics may play a role in the shorter intervention periods and fewer sessions of rehabilitation [58].

The Gauteng Health Strategic Plan 2019/2021–2024/25 highlights the insufficient number of audiologists available to assess patients with chronic illnesses and provide universal hearing diagnostic services for babies as a gap in planning of adequate rehabilitation services, technologies and required hearing devices [39]. It is concerning, yet unsurprising, that audiologists primarily provide services to older adults. A study by Swanepoel et al., which used mobile phone diagnosis for children in the community in South Africa, found a low referral rate of 24.9% for further assessment in preschool children [59]. Additionally, a low follow-up return rate of 39.4% was discovered, primarily due to extended waiting periods before follow-up appointments at PHC clinics. The effective implementation of ear and hearing services at the PHC level requires careful planning, clear programme goals, and defined care pathways [60]. It is important to explore strategies, such as including audiology services into the School Health Programme of the reengineered PHC approach, to enhance the provision of audiology services and increase awareness of their importance. Given the shortage of available services at a limited number of clinics and a national shortage of rehabilitation personnel, especially at PC level a task-sharing approach for a limited set of basic interventions should also be considered to improve access [61].

The number of individual sessions varied across the rehabilitation services, with occupational therapy and speech and language therapy having higher average session numbers compared to physiotherapy and audiology. These variations may reflect differences in treatment protocols, severity of conditions, complexity of the functional outcome or resource allocation. Further research is needed to determine the optimal frequency and duration of rehabilitation sessions to maximise outcomes and patient satisfaction. This research would also inform the packages of care to be funded under the NHI in South Africa.

The findings highlight significant differences in the distribution of rehabilitation services across impairments and age groups [62]. Our study provides an indication of differences in PC rehabilitation services in an urban South African context, considering five rehabilitation services rather than focusing on a single service. This information is valuable for planning rehabilitation services based on disability needs and age-specific patterns. Tailoring rehabilitation services to the unique requirements of people with different disabilities and age groups has the potential to enhance their effectiveness and relevance within UHC provision [2].

An important limitation impacting PC in South Africa is the lack of planning and monitoring, which is essential to inform further development of rehabilitation. The inability to monitor rehabilitation services is due in part to the absence of national agreed minimum data sets for rehabilitation services as well as rehabilitation records and health records in South Africa not being in electronic format. Indeed, the WHO emphasises the functionality of shared electronic health records linked to other health facilities and services to support two-way referrals, multidisciplinary teamwork, and continuity of treatment along clinical pathways [2]. In the current study, the record review was compounded by the variations in record availability and storage among the CHCs and rehabilitation services. Some of the CHCs store their records at a central storage facility while others keep them on-site. The cause of these differences is unknown, but it could be due to a lack of clear record-management policy and storage guidelines, as well as insufficient implementation or resources by rehabilitation professionals to implement existing guidelines. According to the HPCSA, records should be specific, include important information, and should be stored for a minimum of six years from the date of the patient’s last treatment by a healthcare practitioner [63], but the life cycle [64]for other medical records varies for hospitals, health funders and different categories of patients. These discrepancies in record-keeping practicesunderscore the need for a consistent and standardised single record-keeping system across PHC facilities [56]. Additionally, the fact that each rehabilitation service is required to maintain separate records, even during combined sessions for financial and legal/litigation purposes, suggests a lack of integration and siloed service delivery, which affects integration and multidisciplinary teamwork.

Given that the CHCs included in the study serve densely populated and overcrowded townships, where 95% of the population resides, it is crucial to implement integrated and coordinated approaches to rehabilitation service record keeping to ensure continuity of care and integrated service delivery [38].

Strengthening the Routine Health Information System for rehabilitation in the country is needed to allow for regular, easy accessibility and analysis of -real time data compared with population needs, -user profile data, -referral practices and -completion of rehabilitation episodes. This monitoring is essential to plan and evaluate rehabilitation to appreciate the impact of improvement and outcomes [65].

While our study provides valuable insights into the provision of rehabilitation services in the Johannesburg Metropolitan District, it is important to acknowledge some limitations. The retrospective nature of the study and its reliance on the accessibility and accuracy of CHC records may have introduced bias. Moreover, the study’s focus on provided services overlooks those that are not provided and the reasons behind it. Additionally, the study focussed on the nine CHCs offering rehabilitation services, and the findings may not be generalisable to other regions or healthcare settings. Future research should address these limitations and further explore the identified gaps in rehabilitation services to inform policy and practice in a broader context.

## Conclusion

The results from our study outline data available on the rehabilitation services in PHC clinics in the Johannesburg Metropolitan District. The analysis of clinical rehabilitation records from nine provincially funded CHCs between 2011 and 2020 revealed valuable insights into the demographics of clients, types of disabilities, referral sources, service use, and variations in rehabilitation services across the nine CHCs. The findings underscore the diverse rehabilitation needs across different age groups and disabilities and the differences in services offered by different rehabilitation disciplines. Rehabilitation services should be tailored to meet the needs of individuals with a diverse age range, from children to older adults. This includes ensuring services are reflective of all types of care required at different life stages. Missing data revealed the need to improve documentation practices, enhance coordination between healthcare facilities, and expand service delivery models. Future research should explore the alignment of the South African PHC approach with the WHO Package of interventions for rehabilitation [66], the effectiveness of rehabilitation interventions, the impact on patient outcomes, and the factors influencing service provision and access, including the lacking healthcare professions. Strategies to improve the provision and awareness of audiology services should be implemented, especially in the context of the School Health Programme and other community-based initiatives. By addressing these issues, we can strive towards delivering inclusive, person-centred, and integrated rehabilitation services that maximise independence, functioning, and quality of life for individuals in need. Further research using qualitative and mixed method studies are recommended as well as research to develop an agreed minimum rehabilitation dataset for rehabilitation services at the PC level.

## Data Availability

The data that support the findings of this study are available from the corresponding author, LM, upon reasonable request.

## Abbreviations

CHC: Community health centres
NGO: non-governmental organisations
NHI: national health insurance
PC: primary care
PHC: primary healthcare
UHC: Universal Health Coverage
UK: United Kingdom
WHO: World Health Organization
